# Carotid Intima–Media Roughness and Elasticity in Hypertensive Patients With Normal Carotid Intima‐Media Thickness

**DOI:** 10.1002/jum.14843

**Published:** 2018-11-06

**Authors:** Yu Wu, Mingxing Xie, Li Zhang, Xuan Lu, Xinyao Cheng, Qing Lv

**Affiliations:** ^1^ Department of Ultrasound Union Hospital, Tongji Medical College, Huazhong University of Science and Technology Wuhan China; ^2^ Institute of Hematology Union Hospital, Tongji Medical College, Huazhong University of Science and Technology Wuhan China; ^3^ Cardiovascular Division Zhongnan Hospital, Wuhan University Wuhan China

**Keywords:** carotid elasticity, carotid intima‐media roughness, carotid intima‐media thickness, carotid ultrasound, hypertension, vascular ultrasound

## Abstract

**Objectives:**

To investigate carotid intima‐media roughness (IMR) in hypertensive patients with normal carotid intima‐media thickness (IMT) using automatic identification software and the correlation between carotid IMR and risk factors.

**Methods:**

This case‐control study comprised 61 hypertensive patients with normal carotid IMT and 51 control participants. Carotid IMR, carotid IMT, pulsed wave velocity (PWV), stiffness (β), and arterial compliance were determined by carotid ultrasound and image postprocessing using an automatic identification program and echo‐tracking analysis software.

**Results:**

Carotid IMR, mean carotid IMT, maximum carotid IMT, β, and PWV in the hypertension group were higher than those in the control group (58.24 versus 34.61 μm, 641.17 versus 576.48 μm, 746.82 versus 640.55 μm, 9.42 versus 7.35, and 7.10 versus 5.86 m/s, respectively; *P* < .05), and arterial compliance was lower than that in the control group (0.70 versus 0.95 mm^2^/kPa; *P* < .05). Intima‐media roughness was correlated with maximum IMT, mean IMT, PWV, β, age, diagnosis of hypertension for greater than 1 year, and pulse pressure. Multivariate logistic regression showed that age, diagnosis of hypertension for greater than 1 year, and pulse pressure were influential factors for IMR in hypertensive patients, with odds ratios of 6.719 (95% confidence interval, 1.658–27.221; *P* = .008), 4.726 (95% confidence interval, 1.174–19.022; *P* = .029), and 3.998 (95% confidence interval, 1.033–15.466; *P* = .045), respectively.

**Conclusions:**

Carotid IMR and the elasticity index have important clinical importance in evaluating the risk of early atherosclerosis in hypertensive populations.

**Abbreviations**
β: stiffness
BP: blood pressure
IMR: intima‐media roughness
IMT: intima‐media thickness
PP: pulse pressure
PWV: pulsed wave velocity
US: ultrasound

Hypertension has been shown to be clearly associated with cardiovascular disease and mortality.^1^, ^2^ Several studies have shown that carotid intima‐media thickness (IMT) and arterial stiffness in hypertensive patients were significantly higher than those in healthy individuals, and the increase in blood pressure (BP) was closely related to the degree of calcification of the coronary artery and aorta.^3^, ^4^, ^5^ However, there is little information on carotid intima‐media roughness (IMR) in hypertensive patients with normal carotid IMT. Clinically, some patients with hypertension have cardiovascular events before carotid intima‐media thickening. Therefore, early detection of atheromatous changes in hypertensive patients with normal carotid IMT will help a great deal in providing additive information for stratifying patients with cardiovascular risk factors.

Guidelines in China for vascular ultrasound (US) define carotid IMT of 1.0 mm or greater as thickening.^6^ A study by Cheng et al^7^ suggested that carotid IMR increased in high‐risk groups before intima‐media thickening. An automatic identification program was used in this study to measure IMR by calculating the standard deviation of multiple IMT values in the carotid region of interest. The aim of this study was to investigate the relationships between carotid IMR and arterial stiffness and risk factors in hypertensive patients with normal carotid IMT.

## Materials and Methods

### *Patients*

We recruited 61 hypertensive patients with normal carotid IMT. Essential hypertension was diagnosed if systolic BP was 130 mm Hg or higher or diastolic BP 80 was mm Hg or higher or if patients were undergoing treatment with BP‐lowering drugs.^8^ Normal carotid IMT was diagnosed if carotid IMT was less than 1.0 mm by US.^6^

The control group consisted of randomly selected healthy volunteers whose ages and sexes matched the patients in the hypertension group. A total of 51 volunteers were selected for the study. Volunteers with a history of hypertension, diabetes, cerebral infarction, or myocardial infarction were excluded from the study. Electrocardiographic and echocardiographic findings and triglyceride, total cholesterol, low‐density lipoprotein, high‐density lipoprotein, and fasting plasma glucose values were normal in the control group.

This study was approved by the Ethics Committee of the Union Hospital at Huazhong University of Science and Technology, and the methods were applied in accordance with the approved guidelines. Informed consent was obtained from all participants.

### *Ultrasound Examination of the Carotid Artery*

The carotid US examination was performed with an SSD‐α10 US system (Aloka Co, Ltd, Tokyo, Japan) equipped with echo‐tracking analysis software using a UST5412 probe with a transmission frequency of 5–13‐MHz. Image postprocessing used software for automatic segmentation and a thickness uniformity analysis of the carotid intima‐media on US images (National Copyright Administration of China number 2011SR017190).

The participants were placed in a supine position and connected to an echocardiogram. The left common carotid artery within 1.5 cm of the bifurcation was visualized in the longitudinal plane by US. The required image parameters were as follows: (1) the vessel wall was parallel to the probe; (2) the intima‐media layer was clearly visible; (3) the image depth was 4 cm; and (4) the image was frozen at the end of diastole. The image was then imported into the software, and the region of interest was selected. Intima‐media thickness measurements were taken along each vertical pixel line of the region of interest of the artery. The mean IMT was obtained by calculating the average value of these data. Intima‐media roughness was obtained by calculating the standard deviation of these data.

### *Reproducibility of Carotid IMR*

Fifteen randomly selected patients were assessed for interobserver and intraobserver variability of carotid IMR.^9^ To assess intraobserver variability, the same observer measured carotid IMR twice using the software system at an interval of 1 week. To assess interobserver variability, carotid IMR was performed by a second observer, who was blinded to the results of the first observer.

### *Statistical Analyses*

SPSS version 13.0 statistical software for Windows (IBM Corporation, Armonk, NY) was used for statistical analyses. Data were expressed as mean ± standard deviation and numbers of cases. Differences between groups were analyzed by the Student *t* test or Mann‐Whitney *U* test depending on the distribution of the data for continuous variables. The χ^2^ test was used for categorical data. A Spearman correlation analysis was used to explore the relationship between IMR and risk factors. Multiple logistic regression was used for predictors associated with IMR. The Bland‐Altman test was used to show variability in the method. The coefficient of variation was calculated as standard deviation (*x* – *y*)/mean (*x, y*) × 100%. *P* < .05 was considered significant.

## Results

### *Baseline Characteristics*

The 61 hypertensive patients included 36 male and 25 female patients, and their ages ranged from 19 to 77 years. The 51 control participants included 25 male and 26 female participants, and their ages ranged from 29 to 75 years. Among the 61 patients in the hypertension group, there were 36 patients with other risk factors (12 patients with diabetes mellitus, 5 patients with hyperlipidemia, 11 patients with cardiovascular and cerebrovascular diseases, 7 patients with obesity, and 12 patients with smoking); the other 25 patients did not have risk factors.

Diastolic BP, systolic BP, and pulse pressure (PP) in the hypertension group were significantly higher than those in the control group (*P* < .05). There was no significant difference in age, body mass index, fasting plasma glucose level, total cholesterol–to–high‐density lipoprotein ratio, or smoking between the groups (*P* > .05). The results are shown in Table 1.

**Table 1:** Characteristics of Patients and Control Participants

Characteristic	Hypertension (n = 61)	Control (n = 51)	*P*
Age, y	52.30 ± 13.76	48.18 ± 11.20	.089
Body mass index, kg/m^2^	23.98 ± 3.32	22.89 ± 2.67	.063
Systolic BP, mm Hg	146.00 ± 25.95	115.82 ± 6.91	<.001^a^
Diastolic BP, mm Hg	89.64 ± 13.00	75.94 ± 6.82	<.001^a^
PP, mm Hg	56.28 ± 19.05	39.88 ± 6.03	<.001^a^
Fasting plasma glucose, mmol/L	7.29 ± 3.13	6.03 ± 4.18	.072
Total cholesterol/high‐density lipoprotein	3.53 ± 1.90	3.02 ± 0.73	.074
Smoking, n (%)	12 (19.67)	10 (19.61)	.993

a Student *t* test.

### *Ultrasound Examination*

**Figure 1:**
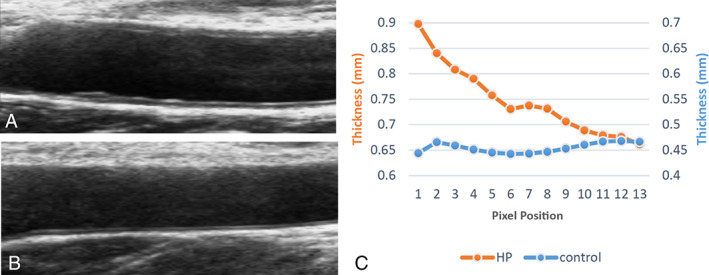
Comparison of smooth and rough intima‐media. **A**, Carotid intima‐media US image from the hypertensive study group. **B**, Carotid intima‐media US image from the control group. **C**, Comparison of the groups. HP indicates hypertensive.

**Figure 2:**
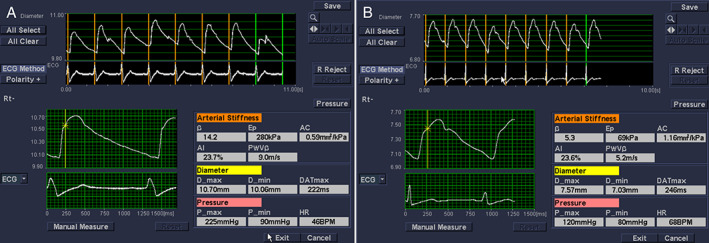
Carotid elasticity index measurements in the hypertension (**A**) and control (**B**) groups. AC indicates arterial compliance; ECG, electrocardiographic; and HR, heart rate. Eρ, pressure strain elastic modulus; AI, augmentation index.

Intima‐media roughness, mean IMT, maximum IMT, stiffness (β), and pulsed wave velocity (PWV) in the hypertension group were higher than those in the control group (*P* < .05; Figure 1); arterial compliance in the hypertension group was lower than that in control group (*P* < .05; Figure 2); there was no significant difference in minimum IMT between the hypertension and control groups (*P* > .05). The results are shown in Table 2.

**Table 2:** Comparison of US Indices Between Hypertension and Control Groups

Parameter	Hypertension (n = 61)	Control (n = 51)	*P*
IMR, μm	58.24 ± 30.47	34.61 ± 18.71	<.001^a^
Mean IMT, μm	641.17 ± 101.91	576.48 ± 108.41	.002^b^
Maximum IMT, μm	746.82 ± 136.97	640.55 ± 123.80	<.001^a^
Minimum IMT, μm	536.37 ± 108.07	513.38 ± 108.37	.265
β	9.42 ± 3.44	7.35 ± 2.74	<.001^a^
PWV, m/s	7.10 ± 1.42	5.86 ± 1.10	<.001^a^
Arterial compliance, mm^2^/kPa	0.70 ± 0.28	0.95 ± 0.36	<.001^a^

a Mann‐Whitney *U* test.

b Student *t* test.

### *Correlation Analysis of Carotid IMR*

Intima‐media roughness was correlated with maximum IMT, mean IMT, PWV, and β (*r* = 0.684; *P* < .01; *r* = 0.451; *P* < .01; *r* = 0.335; *P* < .01; and *r* = 0.333; *P* < .01, respectively). Intima‐media roughness was positively correlated with age, diagnosis of hypertension for greater than 1 year, and PP (*r* = 0.520; *P* < .01; *r* = 0.415; *P* < .01; and *r* = 0.278; *P* < .05). Multivariate logistic regression showed that age, diagnosis of hypertension for greater than 1 year, and PP were influential factors for IMR in hypertensive patients. Age and diastolic BP were influential factors for PWV. The results are shown in Table 3.

**Table 3:** Multivariate Logistic Regression

	IMR ≥ 55.9 μm (30/31)^a^		PWV > 7.0 m/s (31/30)^a^	
Characteristic	OR (95% CI)	*P*	OR (95%CI)	*P*
Age > 50 y (23/38)^b^	6.719 (1.658–27.221)	.008	4.125 (1.138–14.946)	.031
Hypertension > 1 y (34/27)^b^	4.726 (1.174–19.022)	.029		
PP > 50 mm Hg (30/31)^b^	3.998 (1.033–15.466)	.045		
Diastolic BP ≥ 90 mm Hg (30/31)^b^			4.428 (1.230–15.948)	.023

CI indicates confidence interval; and OR, odds ratio.

a Normal/abnormal.

b Young/old or normal/abnormal.

### *Coefficient of Variation of Repeated Measurements*

**Figure 3:**
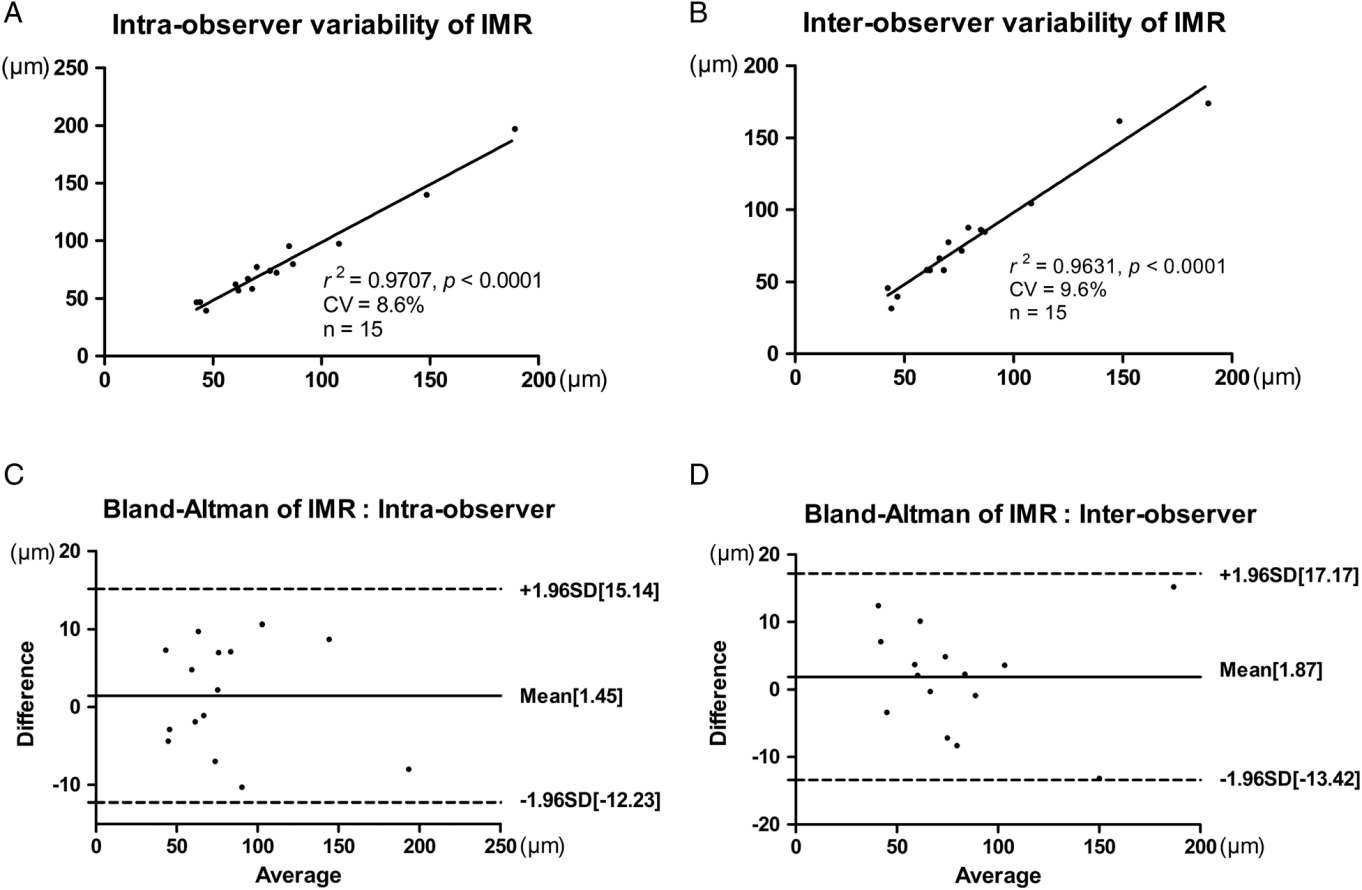
Intraobserver and interobserver variability of IMR measurement. **A**, Intraobserver variability of IMR measurement (n = 15). **B**, Interobserver variability of IMR measurement (n = 15). **C** and **D**, Bland‐Altman plots show that differences between two measurements were similar throughout the range of IMR. CV indicates coefficient of variation.

The measurements of IMR showed intraobserver variability with a coefficient variation of 8.6% (Figure 3A) and interobserver variability with a coefficient of variation of 9.6% (Figure 3B). Bland‐Altman plots show that differences between intraobserver and interobserver measurements were similar throughout the range of IMR (Figure 3, C and D).

## Discussion

Prospective studies revealed that the risk of cardiovascular mortality increases by 15% for every 10‐mm Hg increase in systolic BP in hypertensive patients.^10^ Carotid IMT, as measured by B‐mode US, is a surrogate marker for atherosclerosis.^11^ According to guidelines for vascular US in China,^6^ carotid IMT of 1.0 mm or greater is considered thickening, and 1.5 mm or greater is considered plaque. However, IMT can only reflect the single‐point thickness or average thickness of the intima‐media in arteries, and the morphologic changes of the intima‐media are poorly evaluated. Cheng et al^7^ found that carotid IMR increased in groups with a high risk of cardiovascular disease before IMT. The aim of this study was to investigate the relationships between carotid IMR and arterial stiffness and risk factors in hypertensive patients with normal carotid IMT.

Carotid IMR measured by the software system is consistent with the results of manual measurement in previous studies.^7^ The Bland‐Altman test showed that the measurement parameters were repeatable and could stably reflect the morphologic changes of the carotid intima‐media. Studies by Ishizu et al^12^ and Schmidt‐Trucksäss et al^13^ demonstrated that carotid IMR was significantly increased in patients with coronary heart disease compared with healthy individuals. The results showed that IMR, maximum IMT, and mean IMT in the hypertension group were higher than those in the control group. These findings suggest that in hypertensive patients with carotid IMT of less than 1.0 mm, the IMR is changed, and the intima‐media tends to thicken. This change in the intima‐media layer may be related to the mechanical injury of the arterial wall caused by hypertension and the pathologic characteristics of atherosclerosis. It is known that the changes in pressure caused by hypertension lead to proliferation and hypertrophy of smooth muscle cells and an increase in connective tissue, which, in turn, leads to thickening of the arterial tunica media.^14^ The increase in BP also damages the regulatory barrier function of endothelial cells, which leads to inflammatory factor release, lipid deposition, and other atherosclerotic lesions, resulting in tunica intima thickening.^15^ In atherosclerosis, lipids are deposited unevenly in the intima layer of the artery, and smooth muscle cells and connective tissue proliferate to varying degrees, destroying the smoothness of the arterial tunica intima.^16^ The presence or absence of lesions on the wall and the extent of the lesions may be the main factors leading to IMR changes. Intima‐media thickness cannot distinguish diffuse thickening from focal thickening. The former may be due to the adaptation of the vascular wall to blood flow changes with age. Homma et al^17^ studied 319 healthy people aged 21 to 105 years and found that carotid IMT was linearly related to age, but the plaque incidence increased at 60 to 70 years and then decreased. This finding suggests that carotid IMT is not always consistent with changes in atherosclerosis.

In this study, 55.7% (34 of 61) of the hypertensive patients with normal carotid IMT had a history of hypertension of less than 1 year, and 70.5% (43 of 61) had a history of hypertension of less than 5 years. The IMR, mean IMT, and maximum IMT of patients with a history of greater than 1 year were higher than those of patients with a history of 1 year or less (IMR, 66.88 ± 26.95 versus 51.39 ± 31.72 μm; *P* = .044; mean IMT, 684.37 ± 103.87 versus 606.87 ± 87.42 μm; *P* = .003; and maximum IMT, 799.19 ± 124.69 versus 705.23 ± 133.54 μm; *P* = .006). Multivariate logistic regression showed that a hypertension history of greater than 1 year was a risk factor for IMR. These results suggest that IMR, similar to IMT, is affected by the course of the disease in hypertensive patients, and the risk of atherosclerosis is 4.7 times higher in patients with a diagnosis of hypertension for greater than 1 year than in patients with primary hypertension.

The results showed that β and PWV in the hypertension group were higher than those in the control group, and arterial compliance was lower. It is suggested that the degree of atherosclerosis and elasticity in hypertensive patients with normal carotid IMT are higher than those in healthy individuals. This finding is consistent with previous studies.^18^ In addition, the correlation analysis showed that the elasticity index was positively correlated with the structural index IMR. Stiffness, PWV, and other elasticity indicators mainly reflect the changes in elastin, collagen, and vascular smooth muscle cells and other components in the arterial tunica media.^19^ Intima‐media roughness and IMT mainly reflect intimal atherosclerotic lesions.^16^ There is a relationship between tunica intima and tunica media lesions, yet both have differing characteristics. The results of this study show that age is a common factor for IMR and PWV. The mechanism of the age‐induced decrease in vascular elasticity and structural changes is that vascular wall remodeling, elastic fiber degeneration, collagen fiber increase, and smooth muscle cell proliferation occur under long‐term cyclic tension.^20^ Logistic regression showed that diastolic BP was a factor for PWV but not IMR. It is suggested that the increase in BP can directly decrease the elasticity of blood vessels in hypertensive patients with normal carotid IMT and can indirectly lead to the occurrence and development of atherosclerosis. The reason may be related to direct mechanical stress and injury of the elastic layer of the arterial wall. After mechanical stress injury, the process of atherosclerosis in the arterial tunica intima is accelerated.^21^

In addition, the PP in the hypertension group was significantly higher than that in the control group, and it was an influential factor for IMR. Pulse pressure, as the difference between systolic BP and diastolic BP, is a comprehensive index reflecting systemic vascular elasticity. Increased PP has been shown to result from increased arterial stiffness and decreased compliance.^22^, ^23^ A prospective study of 5991 people who were at least 30 years of age and who were followed for 8.7 years found an increased risk of cardiovascular death of 31% for every 10‐mm Hg increase in PP.^24^ Pulse pressure was also closely associated with atherosclerosis in our study. The reason may be related to alterations of the arterial wall by pulsatile blood flow or cyclic strain, involving not only an endothelial injury of vessels but also an accumulation of collagen fibers and smooth muscle cells within the vascular wall.^25^, ^26^

Our study had several limitations. First, the sample size of this study was limited, and the study did not classify hypertension in different grades and different courses of disease. Moreover, most of our patients were hypertensive for less than 5 years. Additionally, intima‐media analysis software requires a straight, clear lumen in the region of interest to analyze intima‐media information. That cannot be accurately evaluated if there is poor image quality or contouring of the carotid artery. Furthermore, the echo‐tracking technique requires clear and stable images of 5 cardiac cycles to calculate the elastic parameters, resulting in a lack of evaluation for patients with arrhythmia.

In conclusion, carotid IMR was higher and arterial elasticity was lower in hypertensive patients with normal carotid IMT than in healthy control participants. Age is the common influential factor for arterial structure and elasticity. The courses of hypertension and PP are influential factors for carotid IMR. Carotid IMR combined with the elasticity index has important clinical importance in evaluating the risk of early atherosclerosis in hypertensive populations.
